# Clinical utility of contrast‐enhanced ultrasonography in the diagnosis of benign and malignant small renal masses among Asian population

**DOI:** 10.1002/cam4.2635

**Published:** 2019-10-23

**Authors:** Lin Shen, Yanyan Li, Na Li, Yajie Zhao, Qin Zhou, Zhanzhan Li

**Affiliations:** ^1^ Department of Oncology Xiangya Hospital Central South University Changsha Hunan China; ^2^ Department of Nursing Xiangya Hospital Central South University Changsha Hunan China; ^3^ Department of Nuclear Medicine Central South University Changsha Hunan China

**Keywords:** contrast‐enhanced ultrasonography, sensitivity, small renal carcinoma, specificity

## Abstract

We assessed the clinical utility of contrast‐enhanced ultrasonography (CEUS) in the diagnosis of benign and malignant small renal masses using a meta‐analysis of diagnostic test. We performed a comprehensive online search in the following database including PubMed, Embase, Web of Science, Wanfang, and Chinese National Knowledge Infrastructure from the inception to August 25, 2019. the following index were calculated for assessing the diagnostic ability, including sensitivity, specificity, diagnostic odds ratio (DOR), negative likelihood ratio (NLR), positive likelihood ratio (PLR), area under the curve (AUC) with 95% confidence intervals (CIs). Seventeen studies were included in the qualitative and quantitative analyses. The overall sensitivity was 0.93 with 95% CI of 0.88‐0.95. The specificity was 0.71 and the 95% CI was 0.60‐0.80. The pooled AUC was 0.91 (95% CI: 0.88‐0.93). The diagnostic odds ratio was 31 (95% CI: 21‐45). The NLR and PLR were 0.10 (95% CI: 0.07‐0.15) and 3.2(95% CI: 2.3‐4.4), respectively. There is a slight heterogeneity within studies. The subgroup analysis was also performed. For retrospective and perspective, the sensitivity and specificity were 0.93, 0.92 and 0.71, 0.73; For different diameter lesions, the sensitivity and specificity were 0.93, 0.94 and 0.64, 0.74; For sample size (≤median vs. >median), the sensitivity and specificity were 0.94, 0.93 and 0.67, 0.77. The Deek's funnel plot asymmetry test in indicated no publication bias. The CEUS have a high diagnostic ability in differentiating benign and malignant small renal masses among Asian population.

## Graphical abstract

The early detection and timely treatment for small renal carcinoma become one of the most factors in improving curative effect and survival status. The CEUS have a high diagnostic ability in differentiating benign and malignant small renal masses. The CEUS has some characteristics such as non‐invasive operation, relative low cost and no radiation and can be a alternative method for those who cannot take MRI or CT

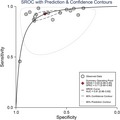

## 1 INTRODUCTION

The small renal masses were defined that the diameter of renal masses measured by enhanced image was equal or less than 4 cm.^1^ It was estimated that 80% of small renal masses were malignant, and most of them were small renal carcinoma.^2^ In the past several decades, the incidence of renal carcinoma was increasing by 2% each year in European and South America.^3^ In the United States, about 30 000 patients were newly diagnosed, and twelve thousand people died of renal carcinoma.^4^ In China, the incidence of malignant tumors is increasing with the changes of genic and environment factors. It was estimated that the incidence of renal carcinoma was 66.8/100 000 and the mortality rate was 23.4/100 000 in 2015.^5^ The renal malignant tumor bring threatens to people's health. The detection rate of small renal carcinoma was being elevated. 25%‐40% of them were detected accidentally.^6^ Most of small renal tumor are at T1a stage. However, some small malignant renal tumors had metastasized at a relatively small volume, and the malignancy gradually increased with the increase in rumor diameter.^7^ Therefore, the early detection and timely treatment for small renal carcinoma become one of the most factors in improving curative effect and survival status.

At present, the methods of detecting small renal masses included computed tomography (CT), magnetic resonance imaging (MRI), conventional ultrasound (CUS), and contrast‐enhanced ultrasound (CEUS).^8^ The CT and MRI have high sensitivity and specificity in detecting renal tumor. However, there are some disadvantages such as ionizing radiation (CT), high examination price and machine cost (MRI).^9^ In some remote region, the MRI was not widely used because of the high machine cost. Ultrasound has been widely used in systemic diseases because of its low cost and equipment. One of its main applications is the examination of kidney and the detection, localization and qualitative diagnosis of neoplastic lesions. However, its important disadvantage is that the detection rate of small renal tumors, especially those less than 2 cm, is lower than that of CT. The CEUS has many advantages such as relative low cost, higher sensitivity and specificity than ordinary ultrasound, and similar detection ability as the CT. Literature on the diagnosis and differential diagnosis of renal benign and malignant masses by contrast‐enhanced ultrasonography is increasing year by year, but most of them are focusing on single symptoms. At present, there is a lack of comprehensive comparative study on the detection ability of contrast‐enhanced ultrasonography for small renal masses with large sample sizes. We performed a systematical search, pooled the extracted data and presented more accurate estimations for the diagnostic ability of CEUS for small renal masses.

## 2 MATERIALS AND METHODS

### 2.1 Search strategy

We performed a comprehensive online search in the following database including PubMed, Embase, Web of Science, Wanfang, and Chinese National Knowledge Infrastructure from the inception to August 25, 2019. We search potential articles in Chinese and English database. We made some combination for the following keywords in the database. These keywords including: (contrast‐enhanced ultrasound OR contrast‐enhanced ultrasonography OR CEUS) AND (renal mass OR renal cancer OR renal tumor OR renal neoplasm OR renal carcinoma OR kidney cancer OR kidney tumor OR kidney neoplasm OR kidney carcinoma OR kidney mass) AND (diagnosis OR diagnostic OR sensitivity OR specificity OR ROC OR receiver operating curve). The references list of included studies was also checked for the potential studies.

### 2.2 Criteria for inclusion and exclusion

The included study must meet the following criteria: (a) a diagnostic screening test of contrast‐enhanced ultrasound for renal carcinoma; (b) The study population focused on small renal carcinoma; (c) The small renal carcinoma was confirmed by pathology examination; (d) the original data (true positive: TP; false positive: FP, false negative: FN; true negative: TN) could be extracted for calculating the diagnostic parameters; (e) for duplicates data, the latest data result was used. Criteria for exclusion: study with animals, without gold standard, with other diagnostic criteria, non‐small renal carcinoma were excluded. We also excluded the reviews, case reports, comments, editorials, letters, and studies without valid data for further analysis.

### 2.3 Data collection process

To keep extracted data accurate, we performed the double extraction plan that two investigators independently collected data and make a cross‐tabulation of the various parameters. We collect the following information for each study: the surname of the first author, publication year, mean age of study population, sample size, the examination machine frequency (MHz), race, gold standard, lesion length, sensitivity, specificity, for folds data (TP, FP, FN, TN).

### 2.4 Assessment of quality

We used the tool of the diagnostic test recommended by the Cochrane handbook, namely quality assessment of diagnostic accuracy studies 2.^10^ This tool consisted of two main domains: risk of bias and applicability concerns. For risk of bias with four items: patient selection, index test, reference standard, and flow and timing. Each item can be judged low risk, high risk and unclear risk. For applicability concerns with three items: patient selection, index test, and reference standard. Each item can be considered as low concern, high concern, and unclear. If any of these items were given high risk or high concern, the study would be judged as high risk of bias.^11^

### 2.5 Statistical analysis

We calculated all diagnostic parameters and plotted the figures using the STATA 14.0 (Stata Corp LP). The plot for quality assessment was performed on Review Manager 5.0 Platform. As no threshold effect existed, we used the bivariate random‐effect model to pool the data in this meta‐analysis.^12^ The heterogeneity was assessed using the *Q* Test and *I* ^2^ statistic. *I* ^2^ ≤ 25% indicated low heterogeneity, 25% < *I* ^2^ ≤ 50% indicated mild heterogeneity, 50% < *I* ^2^ ≤ 75% indicated moderate heterogeneity and *I* ^2^ > 75% indicated high heterogeneity usually indicated and then random‐effect model would be used.^13^, ^14^ For each study we extracted four folds data for analysis including TP, FP, FN, and TN. For all analysis the following parameters were calculated for assessing the diagnostic ability, including sensitivity, specificity, diagnostic odds ratio (DOR), negative likelihood ratio (NLR), positive likelihood ratio (PLR), area under the curve (AUC) with 95% confidence intervals (CIs).^15^, ^16^, ^17^, ^18^ We also conducted the subgroup analysis in study design (retrospective vs. perspective), lesion length (≤3 vs. ≤ 4), and sample size (<62 [sample size median] vs. >62). The sensitivity analysis was evaluated by four ways including goodness of fit, bivariate normality, influence analysis, and outlier detection. The publication bias was assessed using the Deek's plot and relevant statistical test. The diagnostic ability assessment was based the estimated AUC and AUC <0.5 indicated a poor diagnostic ability. *P* < .05 was considered as significant level.

## 3 RESULTS

### 3.1 Study selection and general characteristics

**Figure 1:**
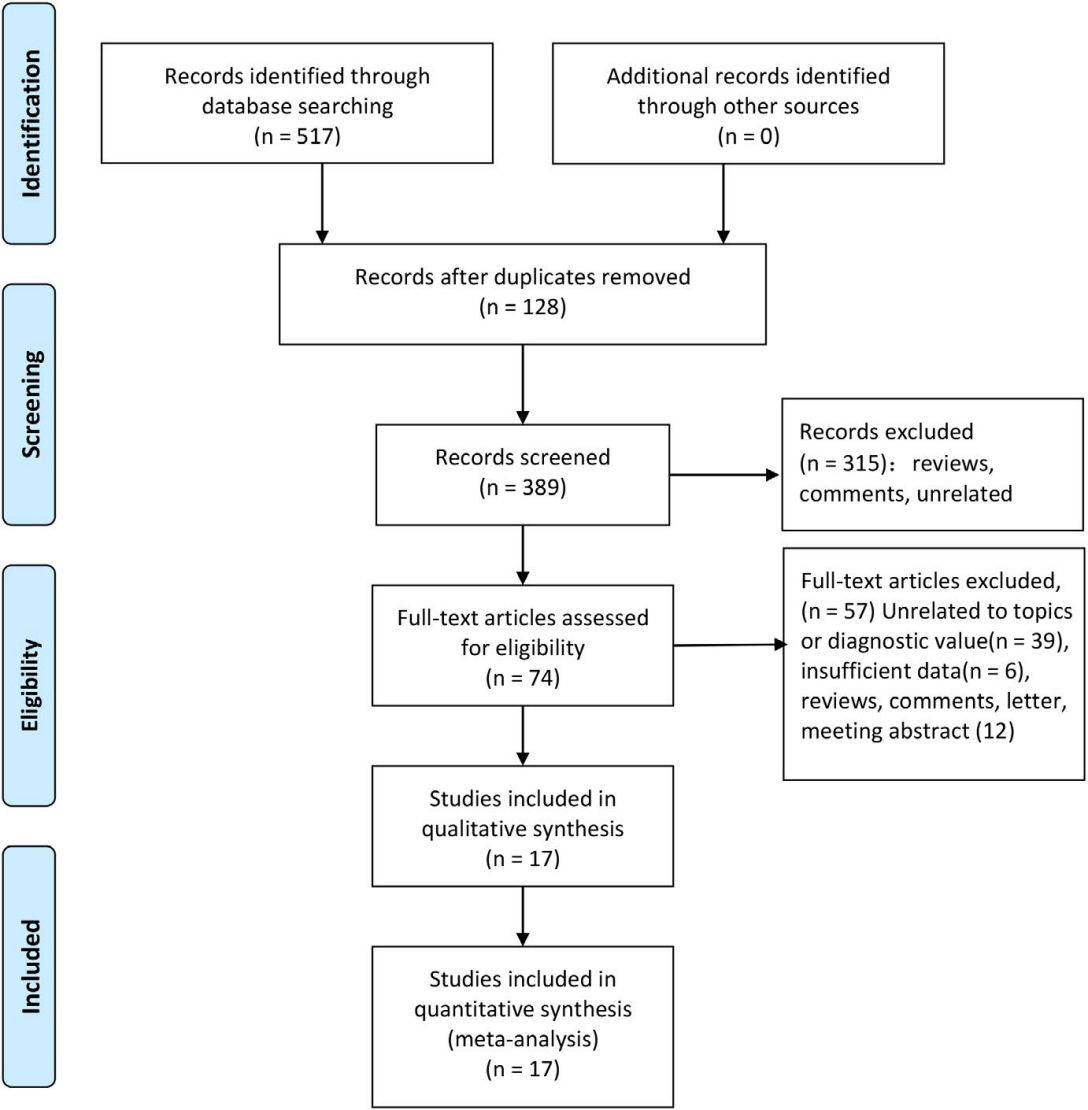
Flow diagram of studies selection process

The Figure 1 presented the flow of study selection. Generally speaking, we obtained 517 records identified through database searching. We did not get additional records identified through other sources. After initial screening, we excluded 128 duplicated records. We further excluded 315 records including reviews, comments obvious unrelated topics from the 389 records. Seventy‐four full‐text studies were assessed for eligibility. Fifty‐seven studies were excluded including 39 studies with unrelated topics or diagnostic value, 6 studies with insufficient data, and 12 other types of studies (reviews, comments, letter, meeting abstract). Finally, 17 studies were included in the quantitative and qualitative analyses (Supplementary material S1).

The Table 1 presented the general detailed characteristics of included studies. Sixteen studies consisted of 11 retrospective studies and 6 perspective studies with 13 070 lesions (30‐145). These studies were published ranged from 2009 to 2019. All studies were from Asian population. The sensitivity ranged from 0.46 to 1.00 and the specificity was from 0.33 to 1.00. Eight studies collected the lesions with ≤3 cm and nine studies collected the lesions with ≤4 cm. The frequency range of machine was between 1.0 and 5.0 MHz. All patients were confirmed by pathology examination.

**Table 1:** General characteristic of included studies

Author	Year	Study design	Age	Machine MHz	Sample size	Race	Gold standard	Lesion length（cm）	TP	FP	FN	TN	Sensitivity	Specificity
Zhang	2017	Retrospective	54.9	1.0‐5.0	95	Asian	Pathology	≤3	74	7	5	9	0.94	0.56
Wang	2018	Retrospective	49.5	3‐5	93	Asian	Pathology	<3	67	3	4	19	0.94	0.86
Lei	2012	Perspective	45.1	3‐4	132	Asian	Pathology	≤3	114	7	5	6	0.96	0.46
Xu	2017	Perspective	56.3	3.5	38	Asian	Pathology	<3	24	1	2	9	0.92	0.80
Li	2009	Retrospective	49.0	3‐5	36	Asian	Pathology	≤4	34	0	1	1	0.97	1.00
Wang	2017	Perspective	55.5	3‐5	145	Asian	Pathology	≤4	73	6	11	28	0.87	0.82
Zhao	2014	Retrospective	46.3	1‐4	54	Asian	Pathology	<3	46	4	1	3	0.98	0.43
Li	2019	Perspective	51.3	1‐5	60	Asian	Pathology	<4	57	2	0	1	1.00	0.33
Gao	2015	Retrospective	59.1	2‐5	32	Asian	Pathology	≤3	18	2	3	9	0.86	0.82
Yan	2016	Retrospective	55.5	2.5‐5	30	Asian	Pathology	<4	23	1	2	4	0.92	0.80
Xue	2019	Perspective	47.5	‐	64	Asian	Pathology	<4	54	2	1	7	0.98	0.78
Li	2011	Retrospective	53.0	4.7‐7.5	72	Asian	Pathology	<4	54	4	4	10	0.93	0.71
Chen	2015	Retrospective	56.6	1‐4	102	Asian	Pathology	<3	73	4	8	17	0.90	0.81
Kang	2016	Perspective	‐	2‐5	96	Asian	Pathology	≤3	76	8	7	5	0.92	0.38
Wei	2017	Retrospective	52.8	1‐5	118	Asian	Pathology	<4	87	8	6	17	0.94	0.68
Oh	2014	Retrospective	61.0	1‐5	49	Asian	Pathology	≤4	33	4	5	7	0.87	0.64
Atrim	2015	Perspective	62.0	1‐5	91	America	Pathology	≤4	21	1	25	25	0.46	0.96

Abbreviations: FN, false negative; FP, false positive; TN: true negative; TP, true positive.

### 3.2 Risk of bias within studies

The quality assessment of included study was presented in the Supplementary material S1 and Supplementary material S2. According to the results, one study was judged as high risk of bias because the index test has a high risk. Two studies have unclear risk of bias in the index test. Three studies have unclear risk of bias in references standard, and on study has unclear risk of bias in the flow and timing. One study has high concern risk. However, the ratio of high risk of bias is less than 10% and the overall quality of included is high.

### 3.3 Synthesis of results

#### 3.3.1 Overall pooled results

**Figure 2:**
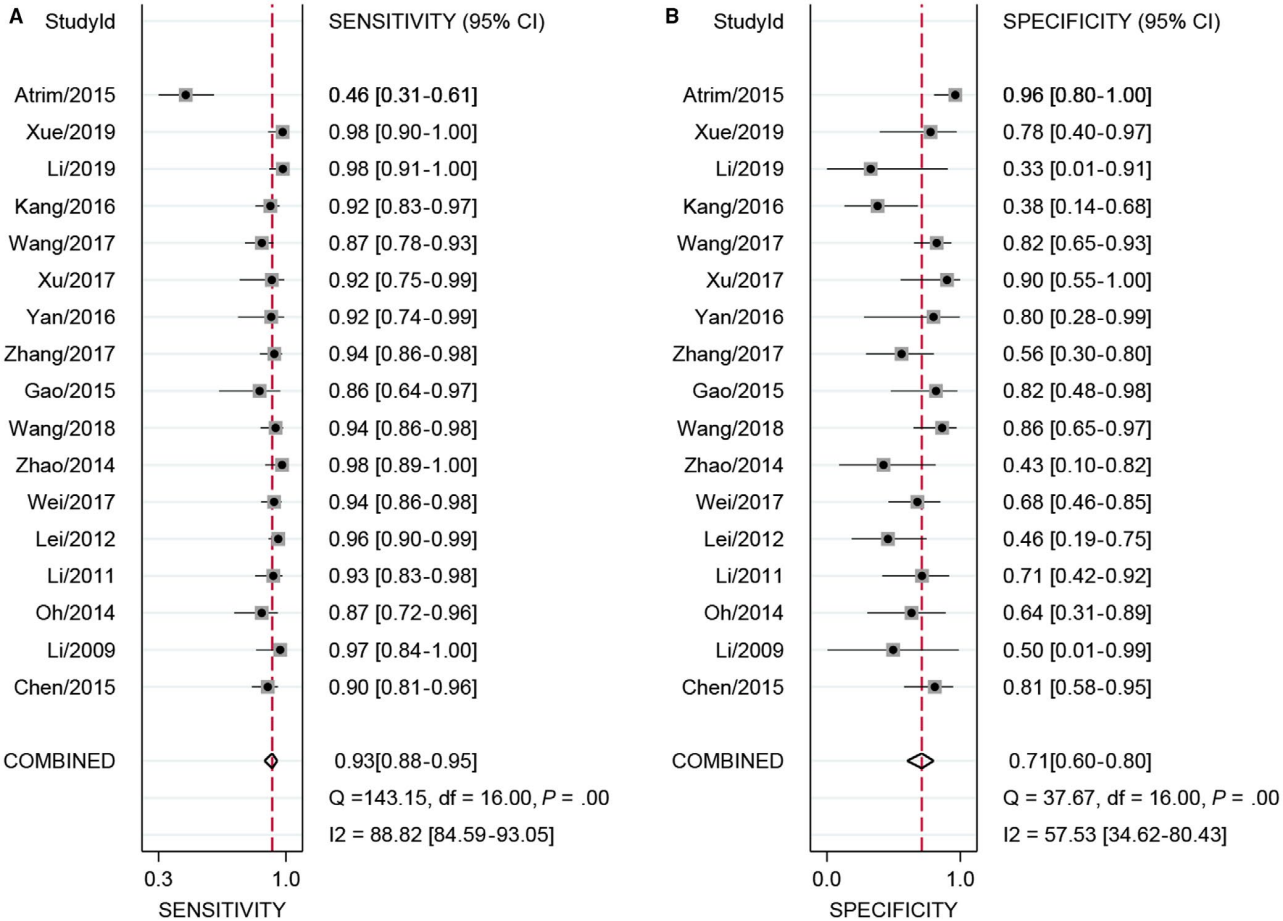
Sensitivity and specificity forest plot of contrast‐enhanced ultrasonography (CEUS) for benign and malignant small renal masses

**Figure 3:**
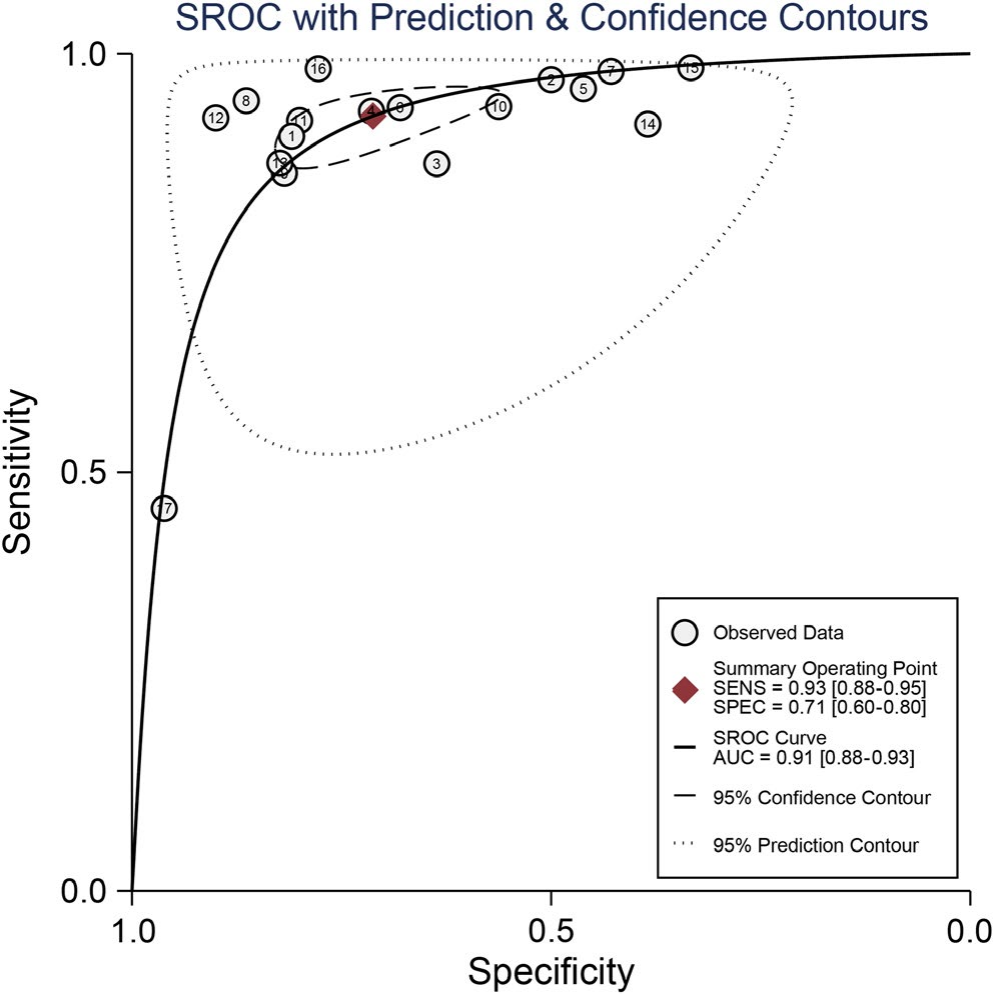
SROC curve of contrast‐enhanced ultrasonography (CEUS) for benign and malignant small renal masses

**Figure 4:**
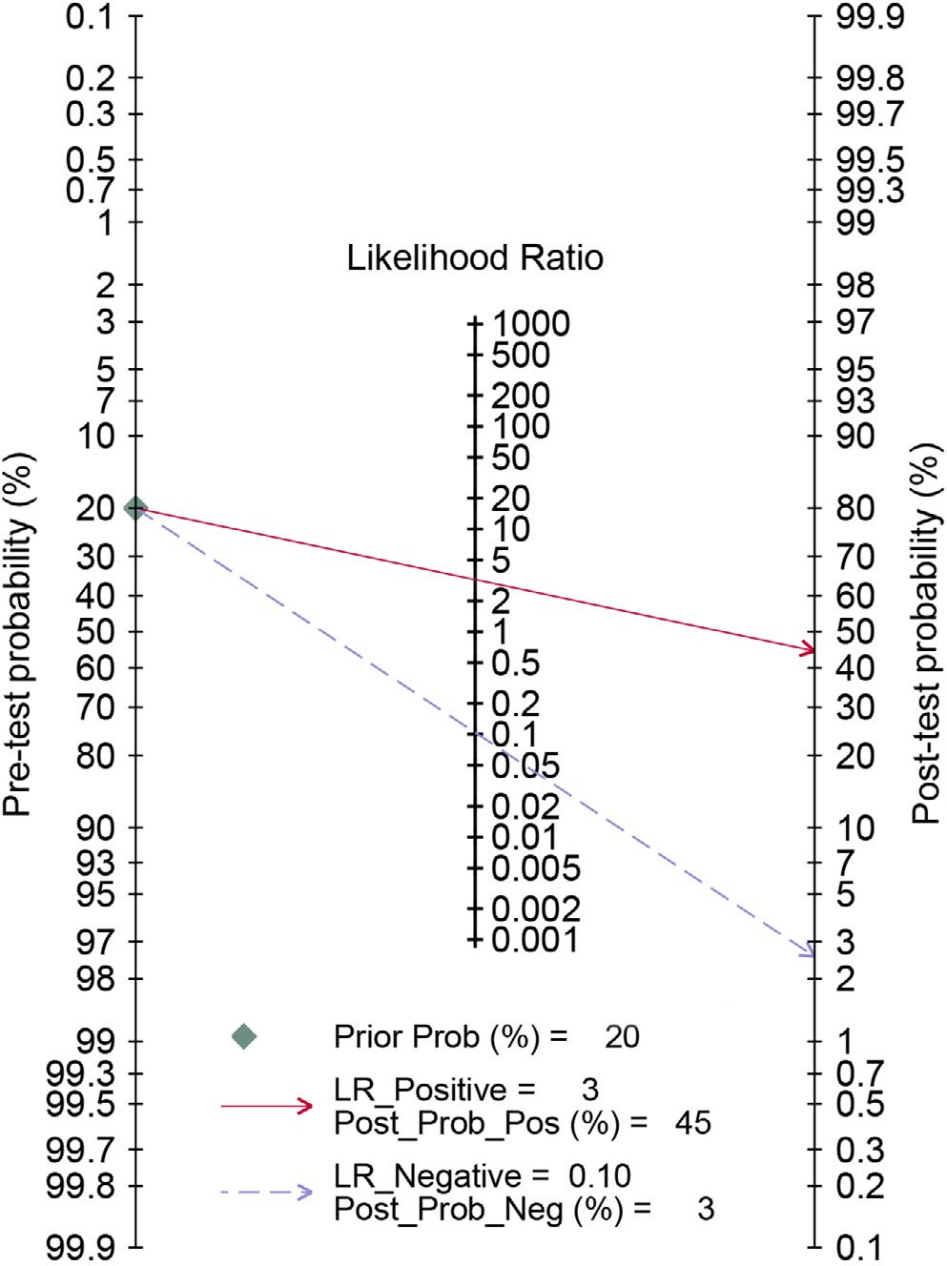
Fagan diagram assessing the overall diagnostic value of contrast‐enhanced ultrasonography (CEUS) for benign and malignant small renal masses

**Figure 5:**
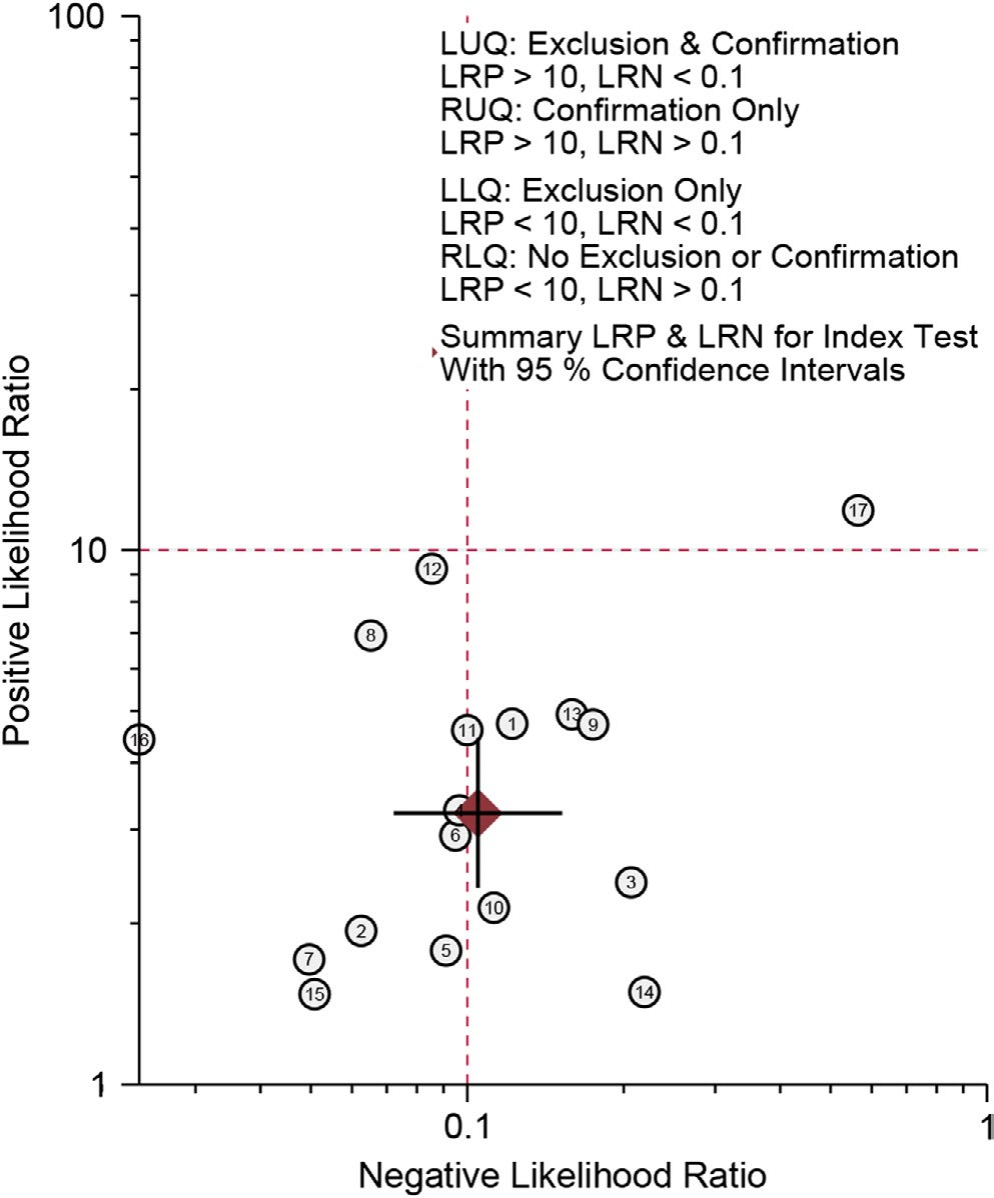
Summary positive likelihood ratio (PLR) and negative likelihood ratio (NLR) for index test with 95% CI

The Spearman correlation coefficient indicated there was no threshold effect. For sensitivity, the heterogeneity test indicated there was no significant heterogeneity (*P* = .00, *I* ^2^ = 88.8%), and the combined sensitivity was 0.93 with 95% CI of 0.88‐0.95 (Figure 2A). The specificity showed a slight heterogeneity (*P* = .00, *I* ^2^ = 57.5%). The pooled specificity was 0.71 and the 95% CI was 0.60‐0.80 (Figure 2B); The Figure 3 presented the summarized receiver operating curve. The pooled AUC was 0.91 (95% CI: 0.88‐0.93). The diagnostic odds ratio was 31 (95% CI: 21‐45). The NLR and PLR were 0.10 (95% CI: 0.07‐0.15) and 3.2(95% CI: 2.3‐4.4), respectively. The Fagan plot was presented in the Figure 4. The pre‐test probability was 20% and the post‐test probability was 45% with the PLR of 3. The Figure 5 presented the summary PLR and NLR for index test with 95% CI. These studies were scattered in the all four quadrants, which means the restricted diagnostic ability. The summary of findings was presented in the Supplementary material S4.

#### 3.3.2 Subgroup analysis

The subgroup analysis was also performed. Eleven studies were based on the retrospective design. The pooled sensitivity was 0.93(95% CI: 0.90‐0.95) and the pooled specificity was 0.71 (95% CI: 0.62‐0.79). There was no heterogeneity within studies (*P* = .197, *I* ^2^ = 0.0%); The ROC area was 0.91 (95% CI: 0.88‐0.93). The diagnostic odds ratio was 3.45(95% CI: 2.97‐3.93). The PLR and NLR were 3.23(95% CI: 2.43‐4.29) and 0.10 (95% CI: 0.07‐0.14). Six studies were based on perspective design. Low heterogeneity was observed (*I* ^2^ = 49.6%). The estimated sensitivity and specificity were 0.92(95% CI: 0.78‐0.97) and 0.73 (95% CI: 0.42‐0.91). The PLR and NLR were 3.40(95% CI: 1.40‐8.40) and 0.11 (95% CI: 0.05‐0.26), respectively. The diagnostic odds ratio was 30.00 (95% CI: 11.00‐78.00). The AUC was 0.92 (95% CI: 0.89‐0.94).

Seven studies focused on these lesions within 3cm. There was medium heterogeneity (*I* ^2^ = 51.4%). The pooled sensitivity was 0.93(95% CI: 0.90‐0.95) and the pooled specificity was 0.64 (95% CI: 0.47‐0.78). The PLR and NLR were 2.58(95% CI: 1.67‐3.99) and 0.11 (95% CI: 0.08‐0.16). The diagnostic odds ratio was 23.52 (95% CI: 12.11‐45.69). The AUC was 0.95 (95% CI: 0.92‐0.96). Ten studies focused on the lesions within 4 cm. Significant heterogeneity was observed (*I* ^2^ = 92.4%). The pooled sensitivity was 0.93(95% CI: 0.85‐0.96) and the pooled specificity was 0.77 (95% CI: 0.65‐0.85). The PLR and NLR were 3.9(95% CI: 2.60‐5.90) and 0.10 (95% CI: 0.05‐0.19). The diagnostic odds ratio was 40.00(95% CI: 22.00‐77.00). The AUC was 0.90 (95% CI: 0.88‐0.93).

Seven studies have sample size within median. There was low heterogeneity within studies (*I* ^2^ = 19.4%). The pooled sensitivity was 0.94(95% CI: 0.89‐0.97) and the pooled specificity was 0.67 (95% CI: 0.48‐0.82). The PLR and NLR were 2.86(95% CI: 1.70‐4.79) and 0.09 (95% CI: 0.05‐0.17). The diagnostic odds ratio was 32.32 (95% CI: 13.77‐75.88). The AUC was 0.92 (95% CI: 0.89‐0.94). Ten studies have sample sizes > median. High heterogeneity was observed (*I* ^2^ = 92.0%). The pooled sensitivity was 0.91(95% CI: 0.85‐0.95) and the pooled specificity was 0.74 (95% CI: 0.60‐0.84). The PLR and NLR were 3.50(95% CI: 2.30‐5.40) and 0.12 (95% CI: 0.07‐0.19). The diagnostic odds ratio was 30.00 (95% CI: 19‐48.00). The AUC was 0.91 (95% CI: 0.88‐0.93).

### 3.4 Sensitivity analysis and publication bias

**Figure 6:**
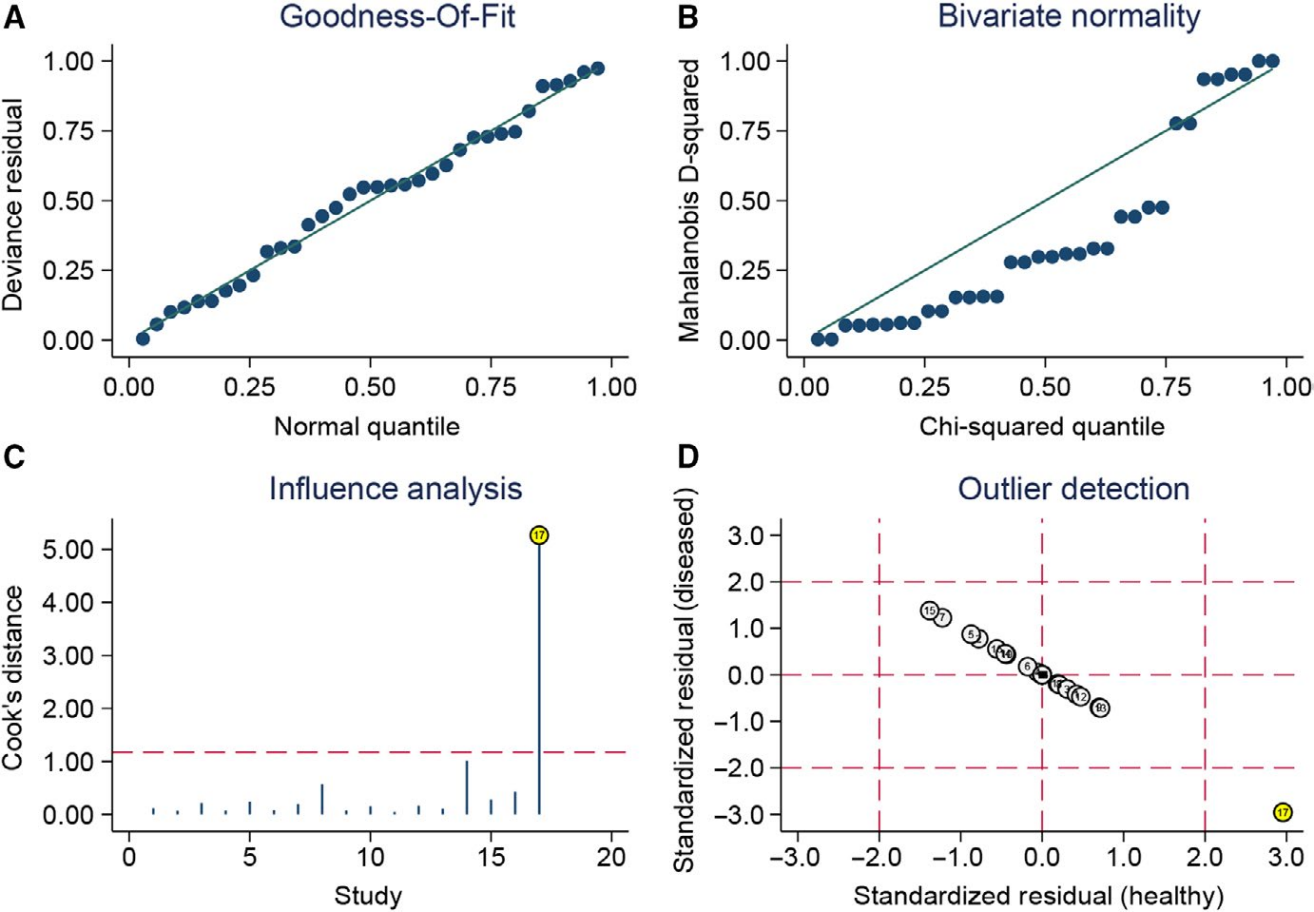
Sensitivity analyses: graphical depiction of residual based goodness‐of‐fit (A), bivariate normality (B), and influence (C), and outlier detection (D) analyses

**Figure 7:**
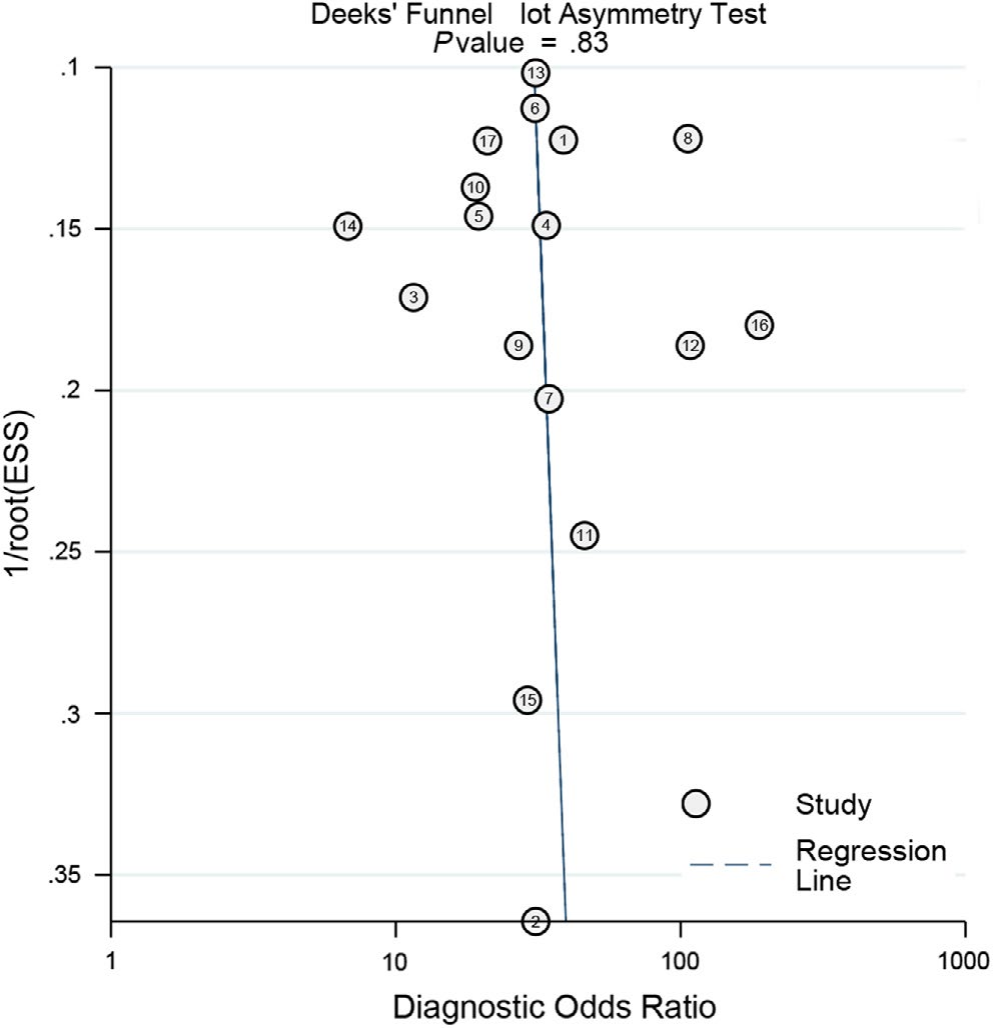
Deeks' plot for Publication bias

The sensitivity analysis is presented in Figure 6A‐D. The goodness of fit (Figure 6A) and bivariate normality (Figure 6B) indicated that the dots basically were scattered on both sides of the straight line. The influence analysis (Figure 6C) suggested the number 14 is outsides of the cook's distance. The outlier detection indicated the number 17 is out of the range (−2 to 2.) The overall pooled results were stable. The Figure 7 gives the assessment of publication bias. The Deek's funnel plot asymmetry test in indicated the funnel plot show no asymmetry (*P* = .830), which means no publication bias existed.

## 4 DISCUSSION

The present results indicated the CEUS has a high sensitivity (0.93) and medium specificity (0.69) in detecting small renal benign and malignant masses. The overall diagnostic ability is high (AUC = 0.93). The diagnostic odds ratio ranged from o to infinity. The higher the DOR is, the higher the diagnosis ability of CEUS is. The pooled DOR was 32 (95% CI: 21‐78) that means the high detection ability. According to the criteria of exclusion and confirmation, PLR >10 and NLR <0.1 indicated high diagnostic ability. The present estimated PLR and NLR were 3.2 and 0.1, which means the CEUS still has some limitations in clinical application. Similar results were found in subgroup analysis. The study design, tumor diameter and sample size could be potential factors of heterogeneity.

In the clinical practice, there are several difficulty points in identifying renal benign and malignant tumor: (a) there are usually no or less fat in the renal hamartoma, the CT imaging of some renal hamartoma is not obvious because of little fat density. When the tumor is mainly composed of smooth muscle and blood vessels and less fat, we cannot get significant CT value, and are easily confused with renal carcinoma; (b) bleeding in the tumor will cover up the examination of fat; (c) When the tumor diameter is less than 1.5cm, the traditional CT and ultrasound examination is not typical due to partial volume effect and respiratory movement; (d) The multitrophic of tumor tissue structure and composition and the atypia of some cells make it difficult to distinguish from renal cancer.^19^, ^20^ The traditional ultrasound examination is inexpensive and widely used in many diseases. One of its main applications is the examination of kidney and the detection, localization and qualitative diagnosis of neoplastic lesions. However, its important disadvantage is that the detection rate of small renal tumors, especially those less than 2 cm, is lower than that of CT.^21^, ^22^ Patients often need to receive enhanced imaging to make definitive diagnosis. Enhanced CT has radiation, and it is difficult to distinguish renal carcinoma from adiposity renal angiomyolipoma.^23^ MRI is recognized as an imaging method with high accuracy in the diagnosis of small renal cancer. However, some patients cannot cooperate due to health conditions and other reasons, such as metal implants in their bodies, or have had MR examination but cannot indicate the exact diagnosis, which limits MR examination.^24^ CEUS can show the real‐time and dynamic perfusion of microcirculation in the lesion, and the contrast agent macrovesicles are discharged through the respiratory tract without liver and kidney toxicity. It is safe to apply in the abdomen and have of great value in differentiating angiomyolipoma from renal carcinoma. Li et al compare the diagnostic accuracy of CEUS and CT in small renal cell carcinoma and found the diagnostic accuracy of contrast‐enhanced ultrasound was 96.67%, which was higher than that of enhanced CT 95.00%. But the difference was not statistically significant.^25^ Oh et al retrospectively assessed the diagnostic accurate in a total of patients with renal masses. The sensitivity and specificity of CEUS were 86.8% and 63.36%, respectively. Their findings were slower than that of the present study.^26^ Wei and his colleagues also compared the diagnostic efficiency of CEUS with that of contrast‐enhanced CT in 118 patients with the small renal masses. The sensitivity and specificity of CEUS were 93.5% and 68%, and the two values of CECT were 89.2% and 76%.^27^ This study indicated that both CEUS and CECT can effectively differentiate diagnostic of benign and malignant small renal masses. The CEUS seems to be more effective than that of CECT. Besides, the reports of this study are similar to our pooled results. The MRI examination has high cost. The examination machine usually is equipped in the senior hospital. MRI examination has gradually become one of the routine examinations. Its high resolution of soft tissue and non‐ionizing radiation have gradually extended its application to the whole body. The examination of kidney disease is one of its main examination items. Gao et al compared the value of CEUS and MRI in the diagnosing of small renal carcinoma. The diagnostic sensitivity and specificity of CEUS were 85.2% and 81.8%, and the sensitivity and specificity of MRI were 90.5% and 90.9%.^28^ Although the diagnostic ability of CEUS was lower than that of MRI, the overall ability was high. The CEUS may be an alternative method for those who cannot take MRI.

Besides, there are inconsistent criteria for the characteristic manifestations of small renal masses. Li et al suggested that the high enhancement during delayed period was an important diagnostic criterion for small renal masses ≤5 cm with the sensitivity of 96.4% and specificity of 77.3%.^29^ Jiang analyzed the CEUS image of renal carcinoma with different size, and thought that renal carcinoma with 2.1‐5.0 cm can be treated as diagnostic parameter.^30^ For the present study, we compared the diagnostic value of CEUS for ≤3 cm and ≤4 cm small renal masses, and found there seemed no significant differences (sensitive: 93.0%, specificity: 64.0% vs sensitivity: 94.0%, specificity: 74.0%). This criterion needs to be further confirmed in the future research. Finally, the CEUS has a good diagnostic ability for small renal masses and also requires some technical skills for operator. The CEUS process involves the close cooperation between the ultrasound scanning doctor and the nurse. The overall process includes the preparation of contrast agent, the establishment of intravenous channels, the injection of contrast agent, postoperative observation and the treatment of adverse reactions. It is very necessary to strictly grasp the indications and contraindications of contrast agents and monitor and deal with adverse reactions in time.

The primary strength is the low heterogeneity within studies (less than 50%) and following the PRISMA checklists. The present studies consisted larger sample size than single study. Several study limitations should be addressed. First, the study population were mainly from Asian population. When new study from America population, the heterogeneity was significantly elevated. But the pooled results were not altered. Further confirmation was required in different study population. Second, we did not include the grey literature and unpublished study data that were usually unavailable. Third, study data were from different regions and affiliations. There may be some differences in evaluating the contrast‐enhanced ultrasonography imaging from different experienced clinicians. This may affect the diagnostic results. Finally, the ultrasonography imaging is different in different pathology type and differentiation degree tumor. We only performed three factors’ subgroup analysis and other potential factors need to be considered in the future studies.

In conclusion, The CEUS have a high diagnostic ability in differentiating benign and malignant small renal masses among Asian population. Compared with CT and MRI, the CEUS has some characteristics such as non‐invasive operation, relative low cost and no radiation. Having considered the study limitation, studies with larger sample size and more accurate design are needed.

## CONFLICT OF INTEREST

The authors declare that they have no conflicts of interest.

## AUTHOR CONTRIBUTIONS

LZZ contributed this idea for the present study. LYY and LN designed the search strategy. ZYJ and ZQ extracted and collected data. LZZ provided analysis software. LZZ and SL drafted the manuscript. SLF revised this manuscript. All authors reviewed this manuscript and approved this submission.

## Data Availability

All data were included in the manuscript and no any restriction for availability.
