# Supplementary material for: Clinical utility of contrast‐enhanced ultrasonography in the diagnosis of benign and malignant small renal masses among Asian population
Source: Cancer Med. 2019 Oct 23;8(18):7532–41. doi: 10.1002/cam4.2635 (PMC6912038; doi:10.1002/cam4.2635)
Supplement: Supplementary file 1 [file CAM4-8-7532-s001.docx]

1. Lei, L., N.H. Fu, B. Yang, and S.P. Wei *et al.* 2012.Comparative analysis of contrast-enhanced ultrasound and contrast-enhanced CT in diagnosis of small renal cell carcinoma. Chin J Imaging Technol. 28: 760-764.

2. Wang, J.Y. *et al.* 2018.Comparative Study of Multi-slice Spiral CT Enhanced Contrast and Contrast-enhanced Ultrasonography in the Diagnosis of Renal Tumors Less than 3 cm. The Prac J Cancer. 33: 675-678+682.

3. Gao, Y.Y., X.P. Yao, L.M. Wang, X.T. Mu, J. Liang, and J.L. Shu *et al.* 2015.Comparison of contrast-enhanced ultrasound and MRI in diagnosis of small renal carcinoma. Chin J Imaging Technol. 31: 758-761.

4. Zhao, J.H., C.Y. Wang, J.Z. Li, Y. Luo, and B. Wen *et al.* 2014.Comparison of diagnosis for contrast enhanced ultrasound and enhanced CT in renal tumors less 3cm. Inter J Urol&Neph. 34: 852-856.

5. Wei, S.P., C.L. Xu, Q. Zhang, Q.R. Zhang, Y.E. Zhao, and P.F. Huang *et al.* 2017.Contrast-enhanced ultrasound for differentiating benign from malignant solid small renal masses: comparison with contrast-enhanced CT. Abdom Radiol (NY). 42: 2135-2145.

6. Oh, T.H., Y.H. Lee and I.Y. Seo *et al.* 2014.Diagnostic efficacy of contrast-enhanced ultrasound for small renal masses. Korean J Urol. 55: 587-92.

7. Xu, J.H., K.F. Fang, Q.Y. Yu, Y. Liu, L. Han, and J. Zhu *et al.* 2017.Diagnostic value of contrast-enhanced ultrasound on small volume renal tumor. Chin J Clin Heal Care. 20: 592-594.

8. Zhang, D., Y.C. Xin, C. Zhang, J. Mu, and Y. Wang *et al.* 2017.Multi-modality imagin in the diagnosis and differential diagnosis of small renal mass. Tianjin Med. 45: 872-876.

9. Kang, S.H., The comparison of diagnosed power in common small renal tumors between magnet resonance imaging andcontrast-enhanced ultrasound. 2016, People's Liberation Army Medical College: Beijing. p. 129.

10. Yan, J.X., The diagnositc of contrast-enhanced ultrasonography in the diagnosis of small renal carcinoma. 2016, Jilin University: Changchun. p. 35.

11. Xue, G.Q. *et al.* 2019.The diagnostic value of color doppler ultrasound in asymptomatic small renal carcinoma. J Imaging Res&Med Appli. 3: 115-116.

12. Chen, L., L. Wang, X. Diao, W. Qian, L. Fang, and Y. Pang *et al.* 2015.The diagnostic value of contrast-enhanced ultrasound in differentiating small renal carcinoma and angiomyolipoma. Biosci Trends. 9: 252-8.

13. Li, Q.J., H.B. Ma, B. Azati, and M. Hothati *et al.* 2019.The Value of Contrast-enhanced Ultrasonography and Contrast-enhanced CT inthe Diagnosis of Small Renal Cell Carcinoma and the Contrast-enhanced Ultrasonography. Med Infromation. 32: 163-165.

14. Li, C.X., X. Yao, X.Y. Li, Y. Xu, Q. Yang, and Z.X. Ye *et al.* 2011.The value of contrast-enhanced ultrasound in the diagnosis of renal tumors equal to or smaller than 4 cm. Chin J Clin Oncol. 38: 520-523.

15. Li, W.G., Y.Y. Zhu, Y. Ruan, J.T. Jiang, Y.Y. Xing, and Y. Shao *et al.* 2009.The value of diagnosis for small renal carcinoma by ultrasonic contrast. J Clin Urol. 24: 32-34+43.

16. Wan, Y.X., J.X. Gao, H.C. Zeng, and L.H. Yao *et al.* 2017.Value of contrast－enhanced ultrasound in diagnosis of benign and malignant nephritic small tumor. J Clin Ultras&Med. 19: 170-173.

17. Atri, M., L. Tabatabaeifar, H.J. Jang, A. Finelli, H. Moshonov, and M. Jewett et al. 2015.Accuracy of Contrast-enhanced US for Differentiating Benign from Malignant Solid Small Renal Masses. Radiology. 276: 900-8.
