# Supplementary figures and images for: Clinical utility of contrast‐enhanced ultrasonography in the diagnosis of benign and malignant small renal masses among Asian population
Source: Cancer Med. 2019 Oct 23;8(18):7532–41. doi: 10.1002/cam4.2635 (PMC6912038; doi:10.1002/cam4.2635)

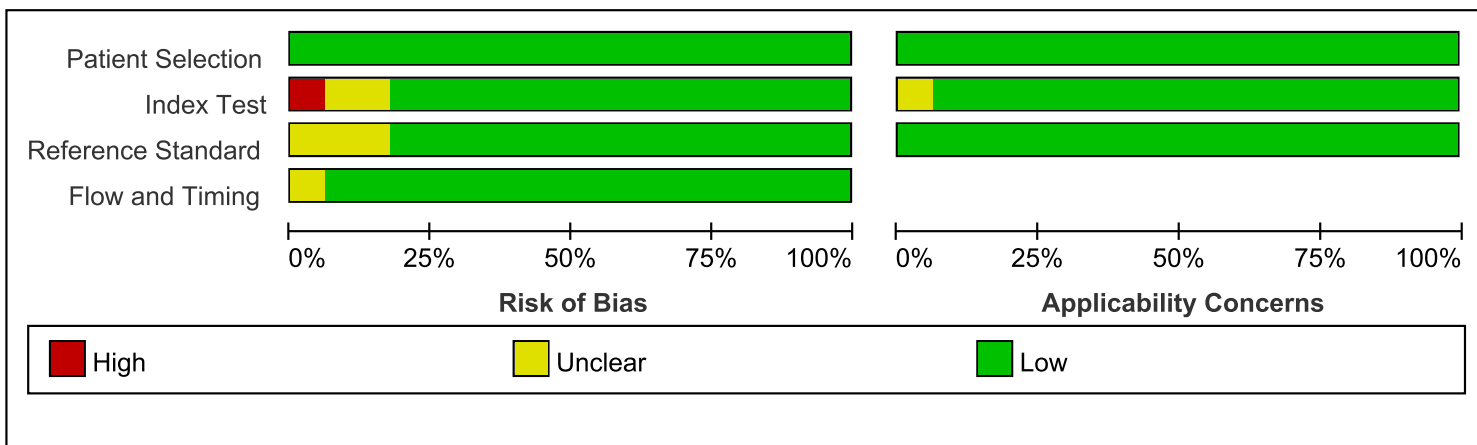

Supplement: Supplementary file 2 [file CAM4-8-7532-s002.pdf]
