# Supplementary material for: Clinical utility of contrast‐enhanced ultrasonography in the diagnosis of benign and malignant small renal masses among Asian population
Source: Cancer Med. 2019 Oct 23;8(18):7532–41. doi: 10.1002/cam4.2635 (PMC6912038; doi:10.1002/cam4.2635)
Supplement: Supplementary file 3 [file CAM4-8-7532-s003.pdf]

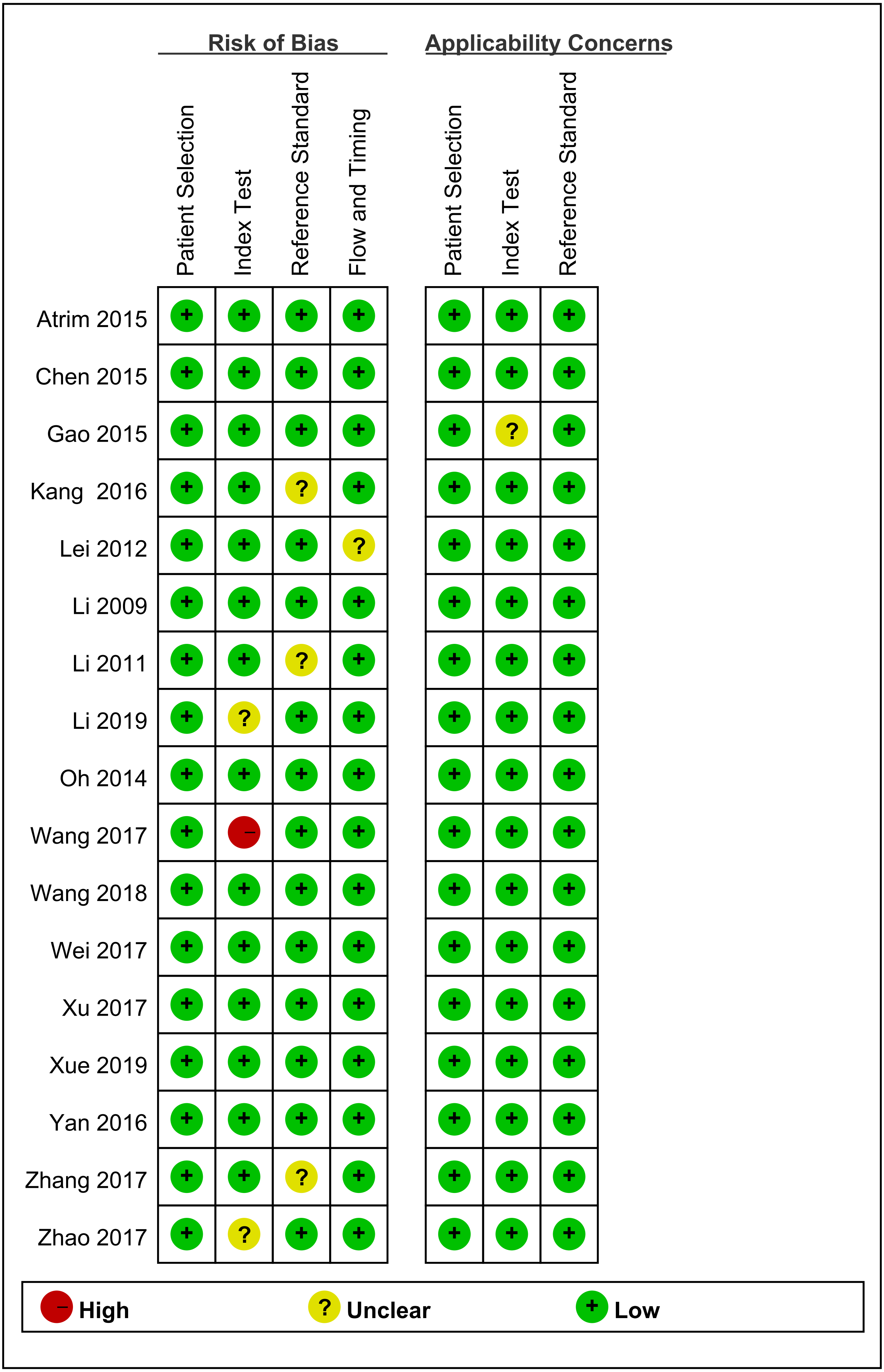
Risk of Bias
Applicability Concerns
Patient Selection
Index Test
Reference Standard
Flow and Timing
Patient Selection
Index Test
Reference Standard
Atrim 2015
Chen 2015
Gao 2015
Kang 2016
Lei 2012
Li 2009
Li 2011
Li 2019
Oh 2014
Wang 2017
Wang 2018
Wei 2017
Xu 2017
Xue 2019
Yan 2016
Zhang 2017
Zhao 2017
High
Unclear
Low
