# Supplementary material for: Clinical utility of contrast‐enhanced ultrasonography in the diagnosis of benign and malignant small renal masses among Asian population
Source: Cancer Med. 2019 Oct 23;8(18):7532–41. doi: 10.1002/cam4.2635 (PMC6912038; doi:10.1002/cam4.2635)
Supplement: Supplementary file 4 [file CAM4-8-7532-s004.pdf]

Question: Should contrast-enhanced ultrasonography be used to diagnose carcinoma in small renal masses?

Sensitivity	0.93 (95% CI: 0.88 to 0.95)
Specificity	0.71 (95% CI: 0.60 to 0.80)

Prevalences	10%	20%	30%

Outcome	№ of studies (№ of patients)	Study design	Factors that may decrease certainty of evidence					Effect per 1,000 patients tested			Test accuracy CoE
			Risk of bias	Indirectness	Inconsistency	Imprecision	Publication bias	pre-test probability of 10%	pre-test probability of 20%	pre-test probability of 30%	
**True positives** (patients with carcinoma)	17 studies 1017 patients	cross-sectional (cohort type accuracy study)	not serious	not serious	not serious	not serious	none	93 (88 to 95)	186 (176 to 190)	279 (264 to 285)	⨁⨁⨁⨁ HIGH
**False negatives** (patients incorrectly classified as not having carcinoma)								7 (5 to 12)	14 (10 to 24)	21 (15 to 36)	
**True negatives** (patients without carcinoma)	17 studies 241 patients	cross-sectional (cohort type accuracy study)	not serious	not serious	serious	not serious	none	639 (540 to 720)	568 (480 to 640)	497 (420 to 560)	⨁⨁⨁◯ MODERATE
**False positives** (patients incorrectly classified as having carcinoma)								261 (180 to 360)	232 (160 to 320)	203 (140 to 280)	
